# Annotations

**Published:** 1886-12-04

**Authors:** 


### Annotations.

**The Analysts on their Defence.**

The Bedford Town Councillor who sent up a sample of poisoned beer to a well-known London analyst has succeeded in raising a hornet's nest, if he has done nothing else. The hornets, however, although they have rallied round their brother and buzzed a great deal, have not buzzed away the hard facts of the case. The sample of beer sent to the metropolis certainly contained a large quantity of deadly poison; as certainly it was tested by an analyst of reputation; and as certainly it was pronounced wholesome. The chemists make a plausible defence for their brother, and they stand by him with one accord, which is very nice and pretty of them. But does that alter the facts one iota? No doubt the public are mere unreasoning barbarians and a hateful *canaille*, whom no person of superior tastes ought to regard with anything but unmixed contempt. Still, that same uncultured public has a habit of trying very hard to look through a milestone, and of seeing the gross facts of a case with unmistakable clearness. Is it really a satisfactory explanation which the analysts with one accord offer? They test, they say, for the ordinary adulterants in any given sample; and if these are absent, they pronounce the sample wholesome. We are anxious to hold a judicial balance between the public and the analysts; but we cannot help feeling that the latter are light in the scale, and unequivocally kick the beam. If their tests are employed only for the ordinary adulterants, then in their pronouncements they are only entitled to declare that these ordinary adulterants are absent or otherwise. Their process must be an exact equation if it is to be regarded as truly scientific, and they must have nothing on the one side which has not its equivalent on the other. When, after such partial testing as they admit they practise, they pronounce a given substance *wholesome*, their conclusion is much larger than their premisses warrant, and as a scientific result is utterly worthless. This will not do: it *must* not do. Science is nothing if it is not exact. The analysts may not be willing to make public confession, but they must be willing to make private and thorough amendment; or if they do not, they will bring themselves and all their works into general contempt.

**Flogging in Board Schools.**

The public excitement about the flogging of Board School children by assistant masters is a "sign of the times." Fifty years ago it would have been thought ridiculous. Were our ancestors wise or are we? There is something to be said on both sides. Perhaps some of the people who are readiest and loudest in offering their opinions are the least entitled to speak. "The heart knoweth its own bitterness," and we suspect that assistant masters in Board Schools know pretty well their own peculiar difficulties and grievances. No doubt flogging is a rough-and-ready method, and equally no doubt it could be almost entirely dispensed with if each pupil could have a teacher to himself. But are our tender-hearted ratepayers prepared to indulge their gentler feelings at such a cost? We must bear in mind that legislation, whether by Parliament or the School Board, is for large numbers, for tens of thousands and even millions; and that those who administer the law as magistrates, policemen, teachers, etc., can only bear a certain proportion to the numbers of those over whom they have control. That being being so, whilst laws and regulations may work very well in the main, there must always be some exceptions, some hardships, some injustice to individuals. This is inevitable, and no system can prevent it. The only question, therefore, that the public should ask is this: "Will it be best on the whole for the discipline and order of the schools that assistant masters should be permitted to use the cane?" If that question be answered in the affirmative no further discussion is necessary or profitable. Let the regulation be fenced round with sufficient safeguards, and then everybody will acquiesce in its wisdom.

**Hydrophobia: Society for its Prevention.**

This is the age of societies, and of secretaries', treasurers', circulars and subscription lists. A man must be possessed of very great courage or of very great stupidity, or have profound faith in his cause, before he will venture to invade our breakfast tables with a circular announcing a new association, especially if he is asking for a large or small amount in the way of a subscription. Paterfamilias may very well be excused if, when he finds that his twenty-fifth circular on Monday morning is a fluent appeal on behalf of the new Taradiddleum Society, he takes his poker, and with grim satisfaction thrusts the circular actually, and at the same time the treasurer and secretary in imagination, into the very midst of the nearest available inferno—the dining-room fire. Still, we are a scientific and a progressive people, and being so we must have societies and associations—even new ones. The philosophical instrument most in request is an intellectual winnowing fan, whereby we may be able to separate the wheat from the chaff—the societies that are really valuable from those that are useless and worse. Making use of such winnowing fan as we possess, we cannot but think there is a necessity for something to be undertaken in the way of preventing the spread of hydrophobia, and that something can probably be taken in hand more efficiently by a society than by any individual, however capable or however leisured. At any rate, such a Society is formed, and shows considerable variety in its composition. It has soldiers, doctors, veterinary surgeons, dog-fanciers, chemists, and university professors among its members. Colonel Rosser is its chairman, Mr. F. G. Debenham its honorary treasurer, and Mr. F. Kerslake its honorary secretary. The names of Prof. Ray Lancaster, Dr. W. R. Gowers, Mr. Victor Horsley, and Prof. Fleming, F.R.C.V.S., are some guarantee that the scientific element will not be wanting. We are glad that the society is not formed for the spread of Pasteurism, but for the far more rational purpose of the prevention and extinction of so terrible a disease as hydrophobia. The offices of the society are at 50, Leicester Square.

